# Regulatory cell therapy for kidney transplantation and autoimmune kidney diseases

**DOI:** 10.1007/s00467-024-06514-2

**Published:** 2024-09-16

**Authors:** Quan Yao Ho, Joanna Hester, Fadi Issa

**Affiliations:** 1 Nuffield Department of Surgical Sciences, University of Oxford, Oxford, Oxfordshire UK; 2 Department of Renal Medicine, Singapore General Hospital, Singapore, Singapore

**Keywords:** Cell therapy, Kidney transplantation, Glomerulonephritis, Tolerance

## Abstract

Regulatory cell therapies, including regulatory T cells and mesenchymal stromal cells, have shown promise in early clinical trials for reducing immunosuppression burden in transplantation. While regulatory cell therapies may also offer potential for treating autoimmune kidney diseases, data remains sparse, limited mainly to preclinical studies. This review synthesises current literature on the application of regulatory cell therapies in these fields, highlighting the safety and efficacy shown in existing clinical trials. We discuss the need for further clinical validation, optimisation of clinical and immune monitoring protocols, and the challenges of manufacturing and quality control under Good Manufacturing Practice conditions, particularly for investigator-led trials. Additionally, we explore the potential for expanding clinical indications and the unique challenges posed in paediatric applications. Future directions include scaling up production, refining protocols to ensure consistent quality across manufacturing sites, and extending applications to other immune-mediated diseases.

**Graphical abstract:**

**Figure:**
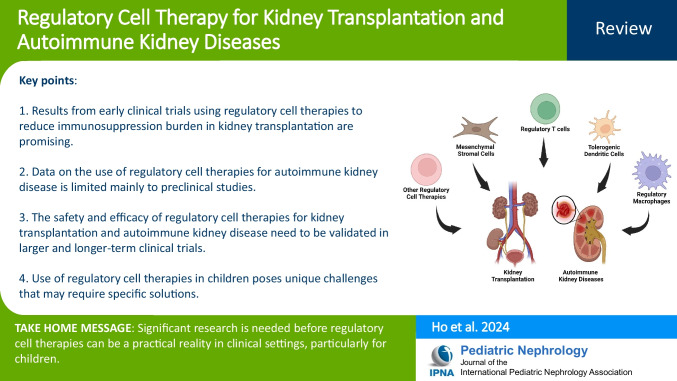
A higher resolution version of the Graphical abstract is available as Supplementary information

**Supplementary Information:**

The online version contains supplementary material available at 10.1007/s00467-024-06514-2.

## Introduction

Regulatory cell therapies represent a cutting-edge approach to the therapeutic modulation of immune responses. These therapies have recently been investigated for their potential to reduce reliance on conventional immunosuppression after kidney transplantation in early clinical trials [1, 2]. Given their immunomodulatory properties, regulatory cell therapies have also been considered for the treatment of other immune-mediated diseases such as graft-versus-host disease (GVHD), inflammatory bowel disease, and autoimmune kidney diseases [3, 4].

This review aims to summarize the current literature on the use of regulatory cell therapies in kidney transplantation and autoimmune kidney diseases, with a focus on strategies with published clinical trials. Pertinent cell biology and preclinical studies will be outlined for each regulatory cell type, together with clinical data and ongoing or planned future clinical trials. Current challenges and directions for future research will be highlighted. While conventional cell therapies targeting B cells have shown some promise for the treatment of autoimmune diseases in recent trials and are discussed in brief, they are beyond the scope of the current review.

## Reducing immunosuppression burden after kidney transplantation

Kidney transplantation significantly enhances survival and quality of life for patients with kidney failure and is substantially more cost-effective than dialysis [5, 6]. However, the advantages of transplantation are curtailed by the long-term side effects associated with immunosuppressive regimens, such as infection, malignancy, and metabolic complications. Improvements in long-term graft survival have remained modest, and chronic rejection continues to be a major cause of graft loss [7]. Innovative approaches to minimise these burdens are critical, particularly given the slow progress in the development of selective pharmacological immunosuppressive strategies. In the ONE study, a phase 1/2A clinical trial, patients treated with 6 different regulatory cell therapy products (Table 1) were compared against standard-of-care (SOC) immunosuppression in living donor kidney transplantation (LDKT). Patients in the cell therapy arms did not receive basiliximab induction with an aim to taper off mycophenolate and minimise tacrolimus dosing. Remarkably, 40% percent of the patients in the cell therapy groups were able to wean to tacrolimus monotherapy while achieving comparable biopsy-proven acute rejection (BPAR) rates and fewer infections. The immune profiles in the cell therapy groups mirrored those of healthy controls, underlying the therapies’ capability to modulate immune responses effectively. Crucially, there were no severe or life-threatening adverse effects attributed to the cell therapies, suggesting a favourable safety profile [1].

**Table 1:** Clinical trials for regulatory cell therapy for kidney transplantation

Cell type	Trial (country)	CTG protocol	Outcomes
ApTregs	TRACT (USA) [11] Phase 1 (non-randomised)	LDKT Alemtuzumab induction Switch TAC to SRL on D30, MMF maintained 500–5000 × 10^6^ cells on D60	*N* = 9 - 1 subclinical rejection with dnDSA - 1 FSGS recurrence with dnDSA
	ONE (Germany) [1, 12] Phase 1/2a (non-randomised with reference control group)	LDKT 0.5–3 × 10^6^ cells/kg on D7 without basiliximab induction TAC trough 3–6 Wk 36, Taper off MMF Wk 48	2 manufacturing failures (inadequate cell numbers) *N* = 11 patients - 8 stable on TAC monotherapy - 2 rejections and 1 IgAN recurrence
	ONE (UK) [1, 2] Phase 1/2a (non-randomised with reference control group)	LDKT 1–10 × 10^6^ cells/kg on D5 without basiliximab induction TAC trough 3–6 Wk 36, Taper off MMF Wk 48	3 manufacturing failures (1 bacterial contamination, 2 inadequate cell numbers) *N* = 12 - 4 stable on TAC monotherapy - No rejection / dnDSA development - Less CMV, BK virus infections
	TASKp (USA) [13] Pilot study	Subclinical rejection on 6-month protocol biopsy in LDKT and DDKT 320 × 10^6^ cells	*N* = 3 - Graft pathology improved in 2 patients but 1 progressed and required corticosteroids
darTregs	ONE / DART (USA) [1, 21] Phase 1/2a (non-randomised with reference control group)	LDKT 0.86–1.9 × 10^4^ cells/kg on D7 to 11 without basiliximab induction MMF tapering on investigator discretion	1 manufacturing failure (inadequate purity) *N* = 3 - 3 stable on TAC monotherapy - No rejection / dnDSA development
MSCs (autologous)	Tan et al. (China) [35] Open-label RCT	LDKT 1–2 × 10^6^ cells/kg BM-MSCs on D0 and D14 without basiliximab induction Corticosteroids, MMF and CNI maintained	*N* = 159 (52 MSC/low dose CNI, 53 MSC/standard dose CNI, 51 basiliximab/standard dose CNI) - Less rejection at 6 months - Less opportunistic infection at 1 year - No difference in kidney function
	TRITON (Netherlands) [36] Open-label RCT	LDKT Alemtuzumab induction 1.5 × 10^6^ cells/kg BM-MSCs at Wk 6 and 7 TAC tapered off Wk 8, EVL maintained	*N* = 29 patients - 1 rejection (vs. 0) - 7 dnDSA development (vs. 2) - No difference in kidney function, IF/TA scores
MSC (third-party)	Erpicum et al. (Belgium) [38] Phase 1/2 (non-randomised with reference control group)	DDKT Basiliximab induction 2 × 10^6^ cells/kg BM-MSCs on D3 MMF and CNI maintained	*N* = 10 - 1 rejection (vs. 0) - 4 dnDSA development (vs. 1)
	NEPTUNE (Netherlands) [39] Phase 1b (non-randomised with reference control group)	LDKT Alemtuzumab induction 1–2 × 10^6^ cells/kg BM-MSC on Wk 25 and Wk 26 TAC reduced to 1.5–3 ng/ml wk26, EVL maintained MSC HLA matched to avoid DSA	*N* = 10 - No rejection / dsDSA development
TolDCs	ONE (France) [46] Phase 1/2a (non-randomised with reference control group)	LDKT 1 × 10^6^ cells/kg on D-1 without basiliximab induction MMF tapering on investigator discretion	1 manufacturing failure (inadequate cell numbers) *N* = 8 - 2 stable on TAC monotherapy - 2 rejection
Mregs	TACI-I (Germany) [53] Phase 1/2	DDKT No antibody induction 1 × 10^6^ cells/kg Taper off SRL Wk 8, TAC reduced by Wk 24, taper off on investigator discretion	*N* = 10 - 8 rejection - 2 tacrolimus monotherapy (withdrew consent)
	TACI-II (Germany) [52] Phase 1/2	LDKT Anti-thymocyte globulin induction 1–10 × 10^7^ cells/kg (Mregs co-cultured with recipient PBMCs) TAC monotherapy by Wk 8, taper off on investigator discretion	*N* = 5 - 3 rejection
	ONE / Mreg_UKR (Germany) [1] Phase 1/2a (non-randomised with reference control group)	LDKT CTP instead of basiliximab reduction Taper off MMF	*N* = 8 Results from subgroup not published
MICs	TOL-1 (Germany) [56] Phase 1	LDKT Donor-derived PBMCs incubated with mitomycin C 1.5–150 × 10^6^ cells/kg on D-2, or 150 × 10^6^ cells/kg on D-7	*N* = 10 - No rejection / dnDSA development

*ApTregs* autologous polyclonal regulatory T cells, *CAR-Tregs* chimeric antigen receptor Tregs, *CMV* cytomegalovirus, *CNI* calcineurin inhibitor, *CTP* cell therapy product, *CTG* cell therapy group, *darTregs* donor antigen reactive Tregs, *DDKT* deceased donor kidney transplant, *dnDSA* de novo donor-specific antibodies, *EVL* everolimus, *LDKT* living donor kidney transplant, *MICs* modified immune cells, *MMF* mycophenolate mofetil, *Mregs* regulatory macrophages, *MSC* mesenchymal stromal cells, *PBMC* peripheral blood mononuclear cells, *RCT* randomised controlled trial, *SRL* sirolimus, *TAC* tacrolimus, *TolDCs* tolerogenic dendritic cells

### Regulatory T cells

CD4 + regulatory T cells (Tregs) are one of the major cell types under investigation for immunomodulation in kidney transplantation. They are characterised by their expression of the transcription factor FoxP3 and play a pivotal role in maintaining immune homeostasis by suppressing auto-reactive immune responses. These cells are characterised by the expression of CD25 and low expression of CD127. Tregs act through multiple mechanisms including the secretion of suppressive cytokines such as TGF-β and IL-10, inhibition of immune cells through contact-dependent mechanisms including CTLA-4 and PD-1, and metabolic disruption of effector T cells through cytokine deprivation, direct cytotoxicity, and ATP degradation [8].

Tregs can be isolated from peripheral blood mononuclear cells (PBMCs) obtained from whole blood or leukapheresis through magnetic-activated (MACS) or fluorescence-activated cell sorting (FACS) and subsequently expanded ex vivo. This expansion typically utilises stimulation with IL-2 and anti-CD3/anti-CD28 beads, often in the presence of immunoregulatory agents like rapamycin to both reduce effector contamination and enhance their suppressive capabilities. Various Good Manufacturing Practice (GMP)-compliant manufacturing protocols have been developed, reflective of the broad interest in their clinical applications [9, 10].

Autologous polyclonal regulatory T cells (apTregs) have been tested in small, non-randomised clinical trials for kidney transplantation. In the TRACT study, 9 patients were given a single, non-weight-based dose of apTregs up to 5000 × 10^6^ cells 60 days after LDKT with alemtuzumab induction [11]. Tacrolimus was converted to sirolimus on day 30 after transplantation, and mycophenolate was maintained. One patient developed subclinical antibody-mediated rejection (ABMR) with the development of de novo donor-specific antibodies (DSAs) at 1 year, while another patient developed de novo DSAs at 1 year with recurrence of focal segmental glomerulosclerosis (FSGS) at 2 years.

In the German arm of the ONE study, apTregs were administered to 11 patients 7 days after LDKT at escalating doses up to 3.0 × 10^6^ cells/kg without basiliximab induction [12]. The aim was to taper off mycophenolate by week 48 while maintaining low dose tacrolimus monotherapy at a trough level of 3–6 ng/ml. Eight patients were stable at 60 weeks post-transplant on tacrolimus monotherapy, while 3 patients experienced acute rejection. Comparatively, in the UK arm of the ONE study, apTregs were administered 5 days after transplant at up to 10 × 10^6^ cells/kg with a similar immunosuppression regimen [1, 2]. No rejection episodes or DSAs were detected, and CMV and BK infection rates were lower in the cell therapy arm. Beyond the initial phases of transplantation, Chandran et al. demonstrated in a pilot clinical trial involving 3 patients that apTregs cells can be manufactured from kidney transplant recipients (KTRs) and safely administered for the treatment of subclinical rejection detected on protocol biopsies [13].

Approximately 10–15% of patients fail to achieve target cell doses during apTregs manufacturing. Risk factors for Treg expansion failure include lower starting cell numbers, reduced proportions of lymphocytes, reduced expression of HLA-DR or CD57 in CD4 T cells, and higher proportions of naïve conventional and regulatory T cells [2, 10]. Identifying patients at risk of Treg manufacturing failure is crucial for efficient resource management, enabling a greater number of patients to benefit from Treg therapy.

Multiple ongoing studies aim to further clarify the role of apTregs in kidney transplantation. The TWO study is an ongoing phase 2b randomised controlled trial (RCT) comparing apTreg cell therapy with reduction of immunosuppression to tacrolimus monotherapy against SOC immunosuppression in preventing BPAR in KTRs [14, 15]. Treg therapy using cells expanded ex vivo using everolimus is being investigated in a pilot study (NCT03284242) [16]. While previous clinical trials have excluded highly sensitised patients, GAMECHANgER-1 is an ongoing phase 2a study testing the effects of apTregs on memory T and B cell responses in sensitised patients on the kidney transplant waitlist [17].

### Treg specificity

Donor antigen-reactive (darTregs) have been shown in pre-clinical studies to be more potent than apTregs and have potentially reduced the risk of unintended immunosuppressive effects since they suppress alloimmune responses in a donor-specific manner [18, 19]. These cells are generated by co-culturing recipient Tregs (or PBMCs which are later sorted for Tregs), with donor cells (e.g. irradiated donor PBMCs, CD40L-stimulated B cells, monocyte-derived dendritic cells) and co-stimulation blockade (e.g. belatacept, anti-CD80/CD86 monoclonal antibodies) [20]. In the ONE study, darTregs were successfully manufactured from 3 of 4 patients and administered between 7 to 11 days after LDKT [21]. These cells were cultured over 72 h with irradiated donor PBMCs and belatacept then isolated through MACS to deplete B cells and CD8 T cells and enrich CD25 cells. The administered dose ranged from 0.86 to 1.9 × 10^4^ cells/kg, which is lower than typical apTreg doses. All patients were successfully weaned to tacrolimus monotherapy, and there were no rejection or severe adverse events attributable to cell therapy. However, the complexity of darTreg manufacturing and the challenges in achieving target cell doses limit their use [22]. The optimal conditions to isolate and expand darTregs are also currently unclear [23]. Moreover, since darTregs require donor cells, their application in deceased donor kidney transplantation (DDKT) may be particularly challenging.

The field is also exploring Tregs engineered to express chimeric antigen receptors (CARs), known as CAR-Tregs. Preclinical studies have demonstrated that CAR-Tregs are more effective than apTregs in mitigating xenogeneic GVHD and rejection in skin and heart transplant [24–26]. The STEADFAST study (NCT04817774), an open-label, phase 1/2a clinical trial, is currently assessing autologous HLA-A2-specific CAR-Tregs produced from HLA-A2 negative patients who are awaiting kidney transplant from HLA-A2 positive donors [27, 28]. However, despite their potential, the high costs, complexity of manufacturing, and variability in HLA matching between donors and recipients pose significant challenges to the widespread adoption of CAR Treg therapy. It is likely that CAR Tregs (or TCR-transgenic Tregs), with targeted specificity against self-presented antigens, will play a crucial role in treating autoimmune diseases. Conversely, in transplantation, the broad direct alloreactivity exhibited by a large percentage of apTregs may be sufficient. Tregs can also switch from an immunosuppressive to a pro-inflammatory phenotype under certain conditions, such as in an inflammatory microenvironment. As such, there is a potential risk that antigen-specific Tregs can undergo such a transition and promote tissue damage. Various strategies to promote phenotypic stability, such as attaching the FoxP3 gene to the CAR transgene insert, are currently under investigation [29]. Nevertheless, these ongoing proof-of-concept trials in transplantation will provide important foundational data.

### Mesenchymal stromal cells

Mesenchymal stromal cells (MSCs) are multipotent, non-haematopoietic cells capable of differentiating into osteoblasts, adipocytes, and chondroblasts and have similarly been investigated as cellular therapy due to their immunoregulatory potential. Characterised by the expression of CD105, CD73, and CD90 and the absence of CD45, CD34, CD14, CD11b, CD79α, CD19, and HLA class II, MSCs modulate immune responses by activating Tregs and other mechanisms such as inhibiting conventional T cell proliferation and polarising macrophages into an M2 phenotype [30–32]. MSCs are typically harvested from plastic-adherent mononuclear cells isolated from source tissues such as bone marrow (BM-MSCs), adipose tissue (AD-MSCs), and umbilical cord blood (UC-MSCs).

In an early feasibility study, two patients treated with MSCs developed acute kidney allograft dysfunction after administration of autologous BM-MSCs 7 days after LDKT, despite an increase in Treg frequency and decrease in CD8 T cell activity. This adverse outcome, attributed to engraftment syndrome caused by the pro-inflammatory polarization of the infused MSCs, led to a revised protocol where MSCs were administered 1 day before transplantation, successfully avoiding allograft dysfunction [33, 34]. Further clinical trials, including a single-centre, open-label RCT of 159 patients, tested 2 doses of autologous BM-MSCs at 1–2 × 10^6^ cells/kg as a replacement for basiliximab induction in LDKT [35]. This trial showed a reduction in BPAR rates at 6 months, as well as fewer opportunistic infections at 1 year in the MSC group. The TRITON study, another RCT involving 70 LDKTs, compared the effects of autologous BM-MSCs administered at 6 and 7 weeks after transplant at a dose of 1.5 × 10^6^ cells/kg with tacrolimus withdrawal at week 8. All but 1 patient in the MSC group successfully weaned off tacrolimus, although there were no differences in kidney function or the interstitial fibrosis score [36]. Donor-derived MSCs have also been previously tested in small uncontrolled studies including one which demonstrated reduced donor-specific reactivity in mixed lymphocyte reaction assays after administration of adipose tissue-derived MSCs isolated from the perinephric fat of living donor kidneys [37].

Allogeneic MSCs from third-party donors are especially attractive since they can potentially be utilised ‘off-the-shelf’. In a phase 1/2 single-centre study by Erpicum et al., 10 patients received third-party BM-MSCs at 2.0 × 10^6^ cells/kg 3 days after DDKT in addition to SOC immunosuppression [38]. Treg frequencies were increased in the MSC group at 30 days, but there was no difference in kidney function at 1 year when compared to a reference control group. Importantly, de novo DSAs against MSC human leukocyte antigens (HLA) developed in 4 patients. In the single-centre, non-randomised, phase 1b NEPTUNE study, 10 patients received 2 doses of allogeneic BM-MSCs at 1.5 × 10^6^ cells/kg at 6 months after LDKT with alemtuzumab induction, followed by reduction of tacrolimus trough levels to 1.5–3 ng/mL [39]. Sensitization against the kidney donor was avoided by matching the HLA of the MSC products. All patients successfully achieved reduced tacrolimus levels without BPAR or de novo DSA formation. Allogeneic umbilical cord-derived MSC administered before and immediately after DDKT as an add-on to SOC immunosuppression failed to demonstrate any benefits in a multicentre RCT that included 42 patients [40].

MSCs have also been explored for the treatment of rejection [41–43]. Six patients with subclinical rejection on a 4-week protocol biopsy or new onset interstitial fibrosis and tubular atrophy (IFTA) on a 6-month protocol biopsy received autologous BM-MSCs without change in maintenance immunosuppression [43]. Immune monitoring suggested a reduced donor-specific immune response and histological evidence of rejection resolving in 2 patients with repeat biopsies, although 3 patients developed opportunistic viral infections. In a study by Wei et al., kidney function over 2 years declined less in 23 patients with chronic ABMR who received allogeneic, third-party BM-MSCs, compared to a retrospective control group [42]. However, another phase 1/2 clinical trial using autologous BM-MSCs with SOC treatment for chronic ABMR was terminated prematurely due to adverse effects and a lack of efficacy [41].

Taken together, the evidence for the efficacy of MSCs in kidney transplantation remains mixed. Early administration before the onset of inflammation and rejection may enhance effectiveness. Ongoing trials are assessing the role of autologous MSCs as an adjunct to SOC immunosuppression in a phase 2 placebo-controlled RCT (NCT03478215), while third-party AD-MSCs administered via the kidney allograft artery are being investigated for the treatment of cellular and ABMR in another phase 1 study (NCT05456243). The evolving landscape of MSC therapy research continues to explore various modifications in the treatment protocols to identify optimal timing and indication.

### Tolerogenic dendritic cells

Tolerogenic dendritic cells (tolDCs) (also known as regulatory DCs, DCregs) are amongst the other cell types investigated for use in kidney transplantation. They are specialised subsets of dendritic cells known for their ability to suppress immune responses and are characterised by low expression of MHC-II and co-stimulatory molecules such as CD80/86 and CD40, increased expression of anti-inflammatory cytokines such as IL-10 and TGF-β, and reduced expression of pro-inflammatory cytokines such as IL-12 [44]. TolDCs inhibit T cell proliferation and induce regulatory cellular responses through several mechanisms including contact-dependent suppression through ICOS-L and PD-L1, the release of immunomodulatory cytokines, and metabolic interferences through the expression of indoleamine 2,3-dioxygenase (IDO) [45].

In the tolDC arm of the ONE study, 8 patients received autologous tolDCs 1 day before LDKT without antibody induction therapy with the option to taper off mycophenolate at a later time point [46]. TolDCs were manufactured by isolating monocytes from recipient PBMCs which were then cultured over 6 days with a low dose (100 U/ml) of recombinant human granulocyte–macrophage colony-stimulating factor (GM-CSF) [47]. Early post-transplant results were mixed; two patients experienced steroid-responsive acute rejection, while another two were successfully weaned to tacrolimus monotherapy.

Donor-derived regulatory dendritic cells have the potential advantage of being able to suppress donor-specific immune responses directly, but equally, there is a risk that they could sensitise the recipient against the donor. To explore these dynamics, a phase 1 clinical trial (NCT03726307) at the University of Pittsburgh is currently assessing the safety of donor-derived, monocyte-derived DCreg therapy as a supplement to SOC in LDKT [48]. Autologous monocyte-derived DCs pulsed with donor antigens have also been shown to prolong graft survival compared to unpulsed DCs in a non-human primate kidney transplant model [49]. TolDCs can be generated through stimulation with cytokines like IL-10 and TGF-β, as well as other small organic molecules. However, the optimal methods for generating these cells remain to be definitively established, reflecting the ongoing research in this field [45].

### Regulatory macrophages

Macrophages are highly plastic and can be polarised by their local microenvironment to adopt an anti-inflammatory and regenerative phenotype [50]. Regulatory macrophages (Mregs) are donor-derived and are generated by culturing mononuclear cells in a medium containing human serum and macrophage colony-stimulating factor, followed by IFN-γ stimulation, and harvesting of the plastic-adherent cells [51–53]. Mregs demonstrate an intermediate phenotype between pro-inflammatory (M1) and regenerative (M2a) macrophages and are characterised by stable upregulation of IDO and have been found to convert allogeneic CD4 T cells to TIGIT-positive induced Tregs [51, 54, 55].

Mregs isolated from the spleen removed concurrently with kidneys from deceased donors were tested in DDKT in the TAIC-I study [53]. Eight of 10 patients who received the cell therapy experienced rejection during immunosuppression withdrawal while the remaining 2 patients withdrew consent while on low-dose tacrolimus monotherapy. In the TAIC-II study, 3 of 5 LDKTs who received Mregs co-cultured with recipient PBMCs developed rejection [52]. In the ONE study, Mregs, referred to as Mregs_UKR, were manufactured from donor CD14 monocytes isolated via MACS [51]. Release criteria for Mregs include CD14, CD16, CD80, CD85h, CD86, CD258, and IDO expression, but the exact specifications are proprietary and not available. Specific results from the Mreg arm of the ONE study are not yet published [1]. The clinical application of Mregs faces challenges, particularly in terms of consistency in therapeutic efficacy and outcomes. The variability in results across different studies indicates a requirement for further work to optimise protocols and assess the conditions in which Mregs can most effectively contribute to reducing immunosuppression.

### Modified immune cells

Mitomycin C can induce non-immunogenic apoptotic cell death and when incubated with donor-derived PBMCs generates ‘modified immune cells’ (MICs), which have been shown to induce tolerance in preclinical studies [56, 57]. In a phase 1 clinical trial, 10 patients received up to 1.5 × 10^8^ MICs/kg before LDKT without induction therapy. No rejection or de novo DSAs were detected, with higher proportions of transitional and memory B cells at follow-up of up to 5 years [58]. A larger, multicentre, open-label phase 2 RCT (NCT05365672) is currently underway to further evaluate the efficacy of MICs against SOC treatment in immunosuppression minimisation [59].

## Autoimmune kidney diseases

**Fig. 1:**
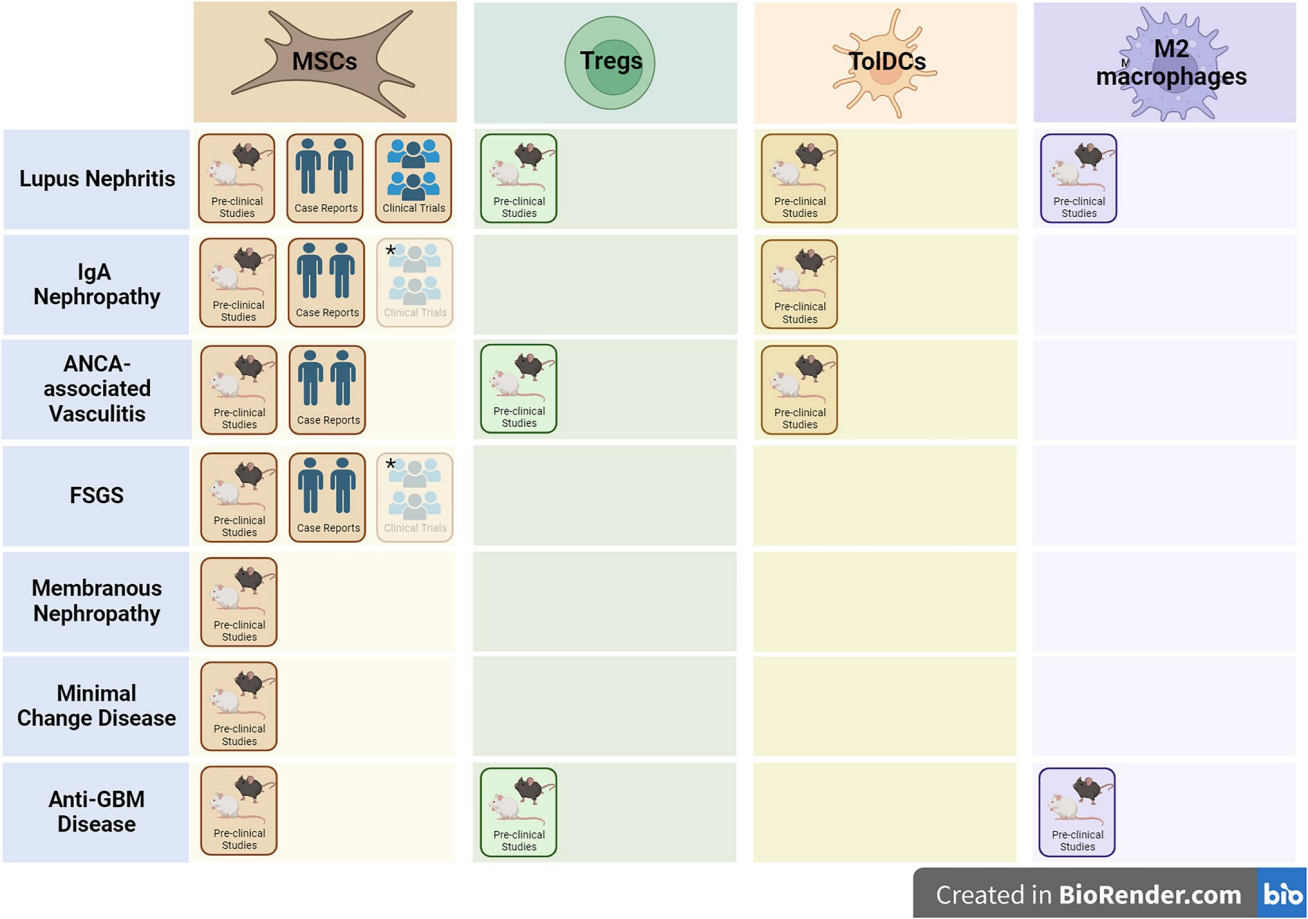
Stage of development for regulatory cell therapy for autoimmune kidney disease. *Trial completed but not yet published. FSGS, focal segmental glomerulosclerosis; anti-GBM disease, anti-glomerular basement membrane disease; MSCs, mesenchymal stromal cells; TolDCs, tolerogenic dendritic cells; Tregs, regulatory T cells

Autoimmune kidney diseases are a heterogeneous group of disorders characterised by an abnormal adaptive immune response against self-antigens and are a major cause of kidney failure in both adults and children [60–62]. Treatments often rely on immunosuppression, which has suboptimal efficacy and poses risks of recurrence and adverse effects. The immunomodulatory potential of regulatory cell therapies makes them promising candidates for treating these conditions, although more clinical studies are needed (Fig. 1). Research in this area may also improve our understanding of the recurrence of autoimmune kidney diseases after kidney transplantation.

### Lupus nephritis

Lupus nephritis, one of the severe manifestations of systemic lupus erythematosus (SLE), is characterised by the development of autoantibodies against nuclear antigens with immune complex deposition and inflammation in the kidneys [63]. B cell-depleting CAR-T cell therapy has been used for severe, refractory SLE in small case series and a phase I clinical trial and was able to achieve drug-free remission in all cases with significant reduction of auto-antibodies at the point of follow-up [64–66]. While these treatments were reported to be well-tolerated without reports of severe cytokine release syndrome (CRS) or immune effector cell-associated neurotoxicity syndrome (ICANS), regulatory cell therapies may potentially offer a safer alternative since they avoid the risks of profound, non-selective B cell depletion.

Autologous BM-MSCs failed to demonstrate any clinical benefit in a case report of 2 patients with SLE [67]. Moreover, BM-MSCs isolated from SLE patients have demonstrated a ‘defective’ and senescent phenotype [68]. Conversely, allogeneic BM-MSCs and AD-MSCs from related donors demonstrated promising results in small, uncontrolled clinical trials for lupus nephritis refractory to standard immunosuppression and as an addition to SOC therapy in a Korean phase 1 clinical trial [69–72]. Unfortunately, an RCT testing allogeneic UC-MSCs as an add-on to first-line SOC immunosuppressive therapy for lupus nephritis was prematurely terminated due to a lack of benefit [73]. Development of anti-HLA antibodies was notably not monitored in any of these studies. Low dose IL-2, for in vivo expansion of Tregs, showed improved response rates compared to placebo in patients with refractory lupus nephritis in 2 RCTs [74, 75]. As ethnicity is known to impact the therapeutic response in lupus nephritis, it is uncertain if these results can be translated to non-Asian populations since most studies were performed in Asia [76].

Several ongoing clinical trials are assessing the use of MSCs for lupus nephritis. The Korean study testing haploidentical allogeneic BM-MSCs has progressed to a phase 2 trial for refractory lupus nephritis (NCT04835883), while a phase 3 RCT in China is comparing UC-MSC against low dose IL-2 as an add-on therapy to SOC immunosuppression (NCT05631717). Outside of Asia, a phase 2 study in Chile is investigating the use of UC-MSCs in severe lupus nephritis (NCT03917797), while another phase 2 study in Spain is assessing allogeneic BM-MSCs for refractory lupus nephritis (NCT03673748).

Beyond MSCs, autologous ex vivo expanded and allogeneic Tregs from umbilical cord blood have demonstrated potential efficacy in preclinical studies, while tolDCs pulsed with histones did not attenuate kidney damage in an SLE mouse model [77–79]. In a recent study, Tregs transduced to express Smith-specific T cell receptors by lentiviral vectors were found to be superior to apTregs in ameliorating disease activity in a humanised lupus nephritis mouse model [80]. Of note, a phase I clinical trial (NCT06265220) utilising allogeneic, off-the-shelf CD19 CAR-NK cells has been initiated.

### IgA nephropathy

IgA nephropathy (IgAN) is characterised by the deposition of immune complexes containing hypo-galactosylated IgA in the kidney, pre-dominantly in the mesangium, leading to variable degrees of inflammation and sclerosis. Clinical presentation ranges from non-visible haematuria to rapidly progressive glomerulonephritis (RPGN) and kidney failure [81]. Importantly, there is no established treatment beyond maximal supportive care, and the efficacy of immunosuppressive agents remains uncertain [82].

BM-MSCs and engineered BM-MSC sheets, hypothesised to be more effective due to the retention of cell adhesion proteins, demonstrated potential efficacy in the anti-Thy1.1 mesangio-proliferative GN rat model [83, 84]. Allogeneic UC-MSC has also been used for severe IgAN in a case report [85]. A phase 1 clinical trial of allogeneic AD-MSC for refractory IgA (NCT04342325) is reported to be complete, but results have not been published [86]. DCs induced to overexpress IDO using a lentivirus vector were shown to reduce kidney IgA deposition, attenuate inflammatory cytokines and cellular response, increase Treg numbers, and reduce urine albuminuria in an IgAN mouse model [87]. Tapping into the potential for cell therapy to fulfil the unmet needs of IgA nephropathy, the Gene-edited T cells combating IgA Nephropathy (GeneTIGA) consortium aims to develop CAR T and TCR-transgenic T cell therapies targeting the B cells producing abnormal IgA [88].

### ANCA-associated vasculitis

Anti-neutrophil cytoplasmic antibody (ANCA)-associated vasculitis (AAV) is characterised by small vessel inflammation frequently involving the kidney with autoantibodies against neutrophil antigens leukocyte proteinase 3 (PR3) or myeloperoxidase (MPO) [89].

Use of MSC-like cells, derived from mature adipocytes, has been described in an AAV mouse model, and autologous BM-MSC was shown to control disease activity for severe kidney AAV in a case report [90, 91]. Odobasic et al. generated antigen-specific tolDCs by culturing bone marrow cells with GM-CSF and an NFκB inhibitor and then pulsing them with myeloperoxidase (MPO). They subsequently demonstrated that tolDCs and the Tregs induced by the tolDCs can attenuate anti-MPO glomerulonephritis (GN) in a mouse model [92]. MPO-specific Tregs which are protective against MPO immunization can also be generated by injecting apoptotic splenocytes conjugated with MPO antigen in a mouse model [93].

### Other autoimmune kidney diseases

Additional autoimmune conditions such as focal segmental glomerulosclerosis (FSGS), membranous nephropathy, and minimal change disease continue to be explored mainly in animal models with various cell therapies [94–106]. Of note, AD-MSCs were found to accumulate in the spleen and not the kidneys when administered intravenously in an anti-GBM (glomerular basement membrane) rat model and were shown to attenuate kidney inflammation by inducing M2 macrophages and Tregs through secretion of extracellular vesicles [106].

## Directions for future research and development

Despite the promising potential to revolutionise kidney transplantation and the treatment of autoimmune kidney diseases, the efficacy and safety of current regulatory cell therapies need confirmation through larger-scale, long-term clinical trials (Table 2). Moreover, current approaches have not been shown to consistently induce operational tolerance. Cell manufacturing failures and the need for complex manufacturing processes may limit their availability and cost-effectiveness.

**Table 2:** Areas of research and development for regulatory cell therapy for kidney transplantation and autoimmune kidney disease

Basic science	Translational and clinical studies	Cell product manufacturing	Implementation
• Improve potency and specificity in future cell products o Different cell types, subtypes, and combinations o Different sources o Selective isolation and expansion o Genetic engineering • Generate allogeneic, ‘off-the-shelf’ therapies o Genetic engineering o In vivo stimulation o Extracellular vesicles	• Confirm efficacy and safety for current indications with large-scale, multicentre studies with longer-term follow-up • Optimise clinical protocols o Patient selection o Timing and dosing o Other immunosuppression o Clinical and immune monitoring • Expand clinical indications o High immunological risk transplants and highly sensitised hosts o Xenotransplantation	• Optimise manufacturing processes – e.g. scalability, reproducibility, automation, and cost • Manage cell manufacturing and expansion failure • Quality control	• Regulatory processes and requirements • Transfer of technology and issues with intellectual property • Logistics—distribution and delivery of manufacturing materials and cell products • Training of healthcare teams • Health economics—cost effectiveness

The ideal cell type or combination for each clinical indication remains undetermined. Additional indications, such as their use in highly sensitised hosts, for the induction of tolerance in xenotransplantation are being explored for existing cell types [17, 107]. Specific subtypes of existing cell products, such as Th2-like Tregs may also be more proliferative and suppressive [108, 109]. Innovations in manufacturing protocols aim to improve the purity, potency, and yield of cell products, such as utilising cells from a different source (e.g. thymic Tregs) or the use of specific compounds (e.g. IL4, IL-5) during the isolation or expansion process [109, 110]. Regulatory B cells, CD8 regulatory cells, and myeloid-derived suppressor cells are also under investigation for their therapeutic potential [111–114].

Cell engineering continues to advance, with developments like the orthogonal IL-2 (ortho IL-2), which selectively stimulates modified IL-2 receptors to maintain function under immunosuppression [115]. Different extracellular binding domains to different members of the TNF-receptor superfamily have also been engineered into Tregs in a CAR construct. These Tregs, known as artificial immune receptor (AIR) Tregs, can home to areas of inflammation and were shown to mitigate GVHD better than control Tregs in a mouse model [116]. Features already built into cell products for haematological malignancies such as hypo-immunogenicity to avoid host-versus-graft disease and allow allogeneic cell products to be used ‘off the shelf’, susceptibility to certain medications which can be used as safety ‘kill switches’, and edited TCRs to avoid GVHD may also be useful for regulatory cell therapies [117, 118].

The optimal timing and dosing for regulatory cell therapies are currently uncertain. Pharmacokinetic studies suggest that Treg therapy can be administered within 2 weeks after anti-thymocyte globulin treatment for most patients, but further research is needed to determine how best to integrate cell therapies with current clinical protocols [119]. Cellular infiltrates, which can be up to 10% Foxp3 positive, have been seen in protocol kidney biopsies in patients receiving Treg therapy [2, 21]. These foci can mimic acute T cell-mediated rejection but do not appear to be associated with adverse outcomes, highlighting the emerging challenges for monitoring KTRs on regulatory cell therapies [120]. Interestingly, similar infiltrates have been recently demonstrated in a spontaneously tolerant mouse kidney transplant model [121].

Optimising GMP manufacturing processes for cell therapies while transferring from the laboratory to an industrial scale is an evolving process. Moreover, to enable widespread use of these regulatory cell therapies in routine clinical practice, GMP cell manufacturing protocols need to be transferred to multiple cell manufacturing facilities while maintaining quality and consistency in the production processes. A number of logistical challenges therefore remain, including navigating multinational regulatory requirements, protecting intellectual property during technology transfer, and improving cost-effectiveness and sustainability within the contexts of different healthcare systems [122, 123].

### Specific considerations in the paediatric population

Translating cell therapies to the paediatric population will pose unique challenges. Cell therapies administered to paediatric patients will need to maintain their efficacy over a prolonged period. Starting immune cell numbers, phenotype and function changes from childhood to adulthood [124]. Over time, Tregs differentiate more from a naïve to an effector memory phenotype, and hormonal changes during adolescence have been shown to alter Treg frequencies and function [125, 126]. How these changes may affect the manufacturing and efficacy of regulatory cell therapies is currently unclear.

Establishing vascular access for paediatric patients to obtain blood for cell manufacturing may also be challenging [127]. Protocols may need to be adjusted to account for children’s lower blood volume and ensure adequate cells can be procured safely [128]. Of note, in a recent phase 2 RCT investigating the efficacy of apTregs for the treatment of children with type 1 diabetes, 15 of 74 patients had to be reassigned to receive a placebo or a lower Treg dose due to their inability to achieve target doses [129]. Regulatory cell therapies may also be unable to prevent recurrent kidney failure due to genetic or congenital disorders which are more common in the paediatric age group [61, 130, 131]. On the other hand, cell therapies have the potential to provide tremendous benefits to paediatric patients. Adverse effects such as growth retardation, chronic infections, loss of immune response to vaccinations, and graft loss due to non-compliance can be avoided by reducing the need for long-term conventional immunosuppression [132, 133].

Unfortunately, clinical trials for regulatory cell therapies for solid organ transplantation in the paediatric group are lacking. THYTECH (NCT04924491) is a phase I/II clinical trial evaluating the safety and efficacy of autologous, thymus-derived Tregs (thyTregs) in preventing rejection in children receiving a heart transplant [110]. ThyTregs are isolated by MACS from thymic tissue collected during heart allograft implantation, expanded under GMP conditions using IL-2 and CD3/CD28 stimulation for 7 days, then administered early post-operatively after patients stabilise from the heart transplant surgery. ThyTregs can be generated in large numbers from thymic tissue which would otherwise be discarded after open-heart surgery and are potentially phenotypically more stable than peripherally isolated Tregs, thereby circumventing multiple challenges with manufacturing Tregs from paediatric patients. A case report of a 7-month-old infant who received thyTregs as part of the trial suggests that ThyTregs are safe and can increase peripheral Treg frequency after 2 years of follow-up [134]. Allogeneic Tregs and Tregs derived from induced pluripotent stem cells which can be utilised ‘off-the-shelf’ can also potentially overcome the challenges of manufacturing cells from paediatric patients.

## Conclusion

While the field holds considerable promise, significant work remains to make regulatory cell therapies a practical reality in clinical settings, particularly for children. Continued preclinical research and careful clinical assessment are essential to overcome the current limitations and fully realise the potential of these innovative treatments.

## Supplementary Information

Below is the link to the electronic supplementary material.

Graphical abstract (PPTX 299 KB)

## Data Availability

Data sharing not applicable to this article as no datasets were generated or analysed during the current study.
