# Supplementary material for: Regulatory cell therapy for kidney transplantation and autoimmune kidney diseases
Source: Pediatr Nephrol. 2024 Sep 16;40(1):39–52. doi: 10.1007/s00467-024-06514-2 (PMC11584488; doi:10.1007/s00467-024-06514-2)
Supplement: Supplementary file 1 — Graphical abstract (PPTX 299 KB) [file 467_2024_6514_MOESM1_ESM.pptx]

## Slide 1
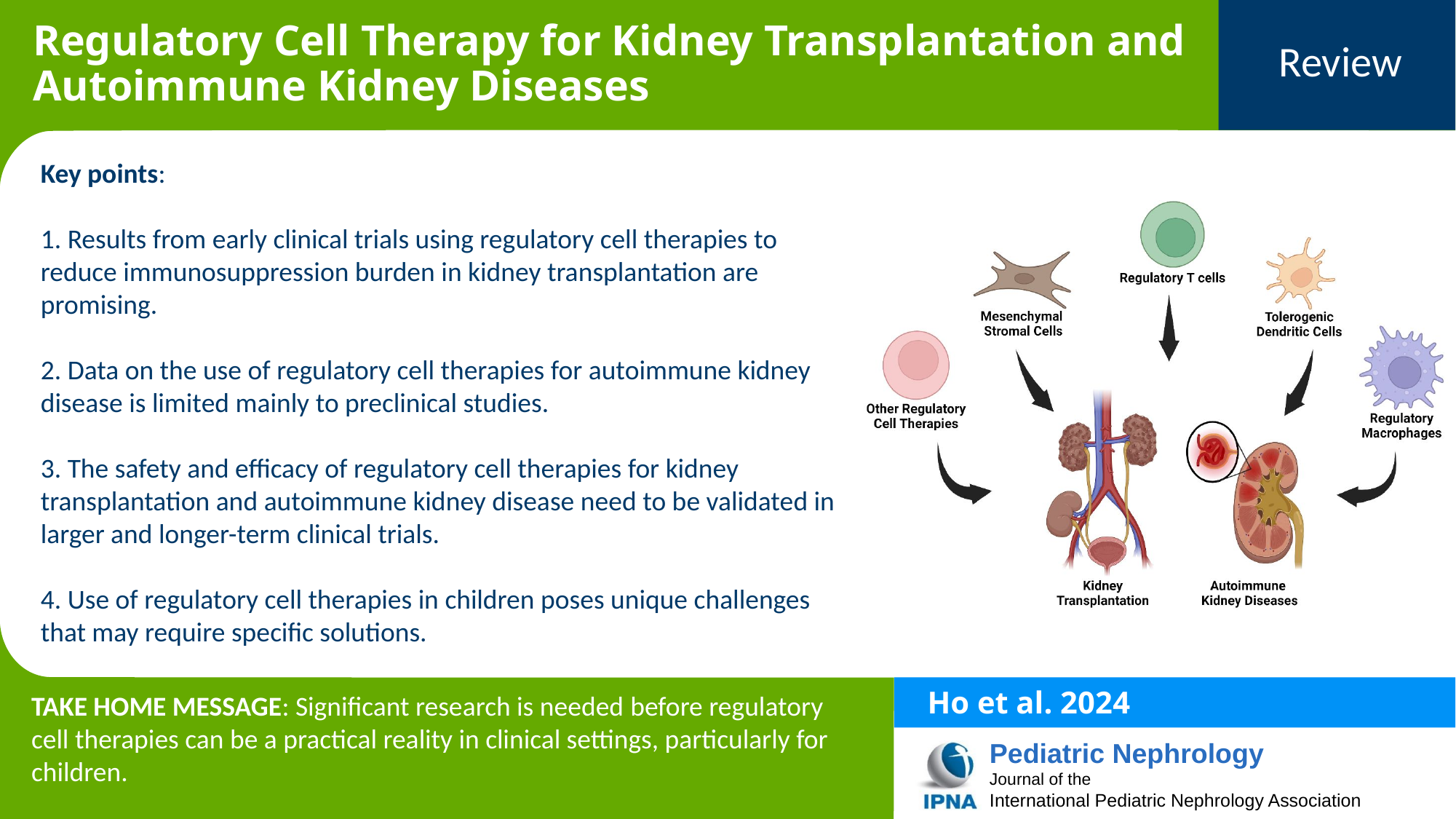

Regulatory Cell Therapy for Kidney Transplantation and Autoimmune Kidney Diseases
Key points:
1. Results from early clinical trials using regulatory cell therapies to reduce immunosuppression burden in kidney transplantation are promising.
2. Data on the use of regulatory cell therapies for autoimmune kidney disease is limited mainly to preclinical studies.
3. The safety and efficacy of regulatory cell therapies for kidney transplantation and autoimmune kidney disease need to be validated in larger and longer-term clinical trials.
4. Use of regulatory cell therapies in children poses unique challenges that may require specific solutions.
Consider including a representative figure or table from your Review article, if relevant, and if you have the requisite permissions.
Ho et al. 2024
TAKE HOME MESSAGE: Significant research is needed before regulatory cell therapies can be a practical reality in clinical settings, particularly for children.
